# Optimizing ceftolozane-tazobactam dosage during continuous renal replacement therapy: some nuances

**DOI:** 10.1186/s13054-019-2724-y

**Published:** 2020-01-10

**Authors:** Gerardo Aguilar, Rafael Ferriols, Sara Martínez-Castro, Carlos Ezquer, Ernesto Pastor, Jose A. Carbonell, Manuel Alós, David Navarro

**Affiliations:** 1 Critical Care Unit, Anesthesiology and Critical Care Department, Hospital Clínico Universitario de Valencia, Valencia, Spain; 2 INCLIVA Health Research Institute, Avenida de Menéndez y Pelayo, 4, 46010 Valencia, Spain; 3 School of Medicine, University of Valencia, Avenida Blasco Ibáñez, 15, 46010 Valencia, Spain; 4 Department of Pharmacy, Hospital Clínico Universitario de Valencia, Avenida Blasco Ibáñez, 17, 46010 Valencia, Spain; 5 Department of Microbiology, Hospital Clínico Universitario de Valencia, Avenida Blasco Ibáñez, 17, 46010 Valencia, Spain

We have read the recent letter by Honore et al. [1] about our findings published in this journal regarding the influence of continuous renal replacement therapy (CRRT) on the pharmacokinetics of ceftolozane-tazobactam (C/T) [2]. In our report, we decided to administer a 3 g/iv dose every 8 h taking into account two previous studies referenced in our paper [2] and another one which showed CRRT to be an independent predictor of clinical failure (OR 4.5, 95% CI 1.18–17.39, *p* = 0.02) when C/T is administered at 1.5 g every 8 h [3].

As Honore et al. explain in their paper, the C/T eliminitation was assumed by hemodiafiltration and the adsorption was not assessed [1]. However, there is a misunderstanding in this letter [1], because we used a polysulphone membrane (Fresenius, Germany) instead of an acrylonitrile 69 Multiflow (AN-69-M). In contrast to highly adsorptive membranes (HAM; e.g., AN69 surface-treated, AN69-ST), the antibiotic adsorption with polysulphone ones is negligible, which facilitates antibiotic adaptation during CRRT [4].

Our data should not be extrapolated to other clinical scenarios, as noted by Honore et al. [1]. In our report, ceftolozane and tazobactam plasma concentrations remained above the minimal inhibitory concentration (MIC), for MICs of up to 8 μg/mL, but we estimated that the administration of standard doses of 1 g/0.5 g, even with polysulphone membranes, could compromise the effectiveness of C/T for not reaching adequate tazobactam concentrations. Thus, the use of HAM would represent a real risk factor of clinical failure when a C/T dose of 1.5 g every 8 h is administered, especially in multidrug-resistant infections [3]. Therefore, we agree with Honore et al. [1] that therapeutic drug monitoring (TDM) is critical when using C/T for patients receiving CRRT, especially when MICs of bacteria like multidrug-resistant (MDR) *Pseudomonas aeruginosa* are considered very high. However, the recommendation of continuous (over 24 h) vs extended (over 2 to 4 h) or intermittent (over 30 to 60 min) infusion of beta-lactams is still under debate [5].

## Data Availability

Not applicable.

## Abbreviations

AN-69-M: Acrylonitrile 69 Multiflow
AN-69-ST: AN69-surface treated
C/T: Ceftolozane-tazobactam
CRRT: Continuous renal replacement therapy
HAM: Highly adsorptive membranes
MDR: Multidrug-resistant
MIC: Minimal inhibitory concentration
